# Draft Genome Sequence of *Lactobacillus plantarum* MPL16, a Wakame-Utilizing Immunobiotic Strain Isolated from Swine Feces

**DOI:** 10.1128/genomeA.00006-17

**Published:** 2017-03-09

**Authors:** Julio Villena, Lucila Saavedra, Elvira Maria Hebert, Yoshihito Suda, Yuki Masumizu, Leonardo Albarracin, Patricia Clua, Wakako Ikeda-Ohtsubo, Haruki Kitazawa

**Affiliations:** a Reference Centre for Lactobacilli (CERELA-CONICET), San Miguel de Tucumán, Tucumán, Argentina; b Food and Feed Immunology Group, Laboratory of Animal Products Chemistry, Graduate School of Agricultural Science, Tohoku University, Sendai, Japan; c Department of Food, Agriculture and Environment, Miyagi University, Sendai, Japan; d Livestock Immunology Unit, International Education and Research Center for Food Agricultural Immunology (CFAI), Graduate School of Agricultural Science, Tohoku University, Sendai, Japan

## Abstract

The genome of the immunomodulatory *Lactobacillus plantarum* MPL16, a strain able to ferment wakame (*Undaria pinnatifida*), is described here. The reads were assembled into contigs with a total size 3,278,495 bp. The genome information will be useful for further specific genetic studies of this strain that evaluate its immunomodulatory and biotechnological properties.

## GENOME ANNOUNCEMENT

Gastrointestinal infections in weaning pigs are a major global problem (1). Several alternatives are being studied to improve the resistance of pigs against gut pathogens. It was reported that the feeding of the seaweed wakame (*Undaria pinnatifida*) augmented mucosal immune functions in pigs (2). We hypothesized that a combination of wakame and immunomodulatory lactobacilli able to metabolize and grow in this seaweed could be used as a superior functional feed to improve porcine gut immunity. Therefore, we isolated lactobacilli from swine intestine and feces and evaluated their capacity to utilize wakame for growing, as well as their immunomodulatory activities in porcine intestinal epithelial (PIE) cells. A *L. plantarum* strain isolated from feces, named MPL16, showed a higher capacity to grow utilizing wakame and modulate the production of inflammatory cytokines in PIE cells (Masumizu et al., unpublished data).

Here, we present a draft genome sequence of *L. plantarum* MPL16, which was sequenced using a whole-genome shotgun strategy on an Illumina MiSeq sequencer. Paired reads with lengths of 300 bp were obtained corresponding to a 3,580-fold coverage. Quality-filtered reads were assembled using Ngen version 12.2.0 software (DNASTAR). The RAST server and the NCBI’s Prokaryotic Genome Annotation Pipeline were used for the functional annotation of predicted genes (3). tRNAs and rRNAs were identified by tRNAscan-SE and RNAmmer, respectively (4, 5).

The draft genome of *L. plantarum* MPL16 consists of 3,278,495 bp with a mean G+C content of 43.6%. A total of 2,880 coding sequences (CDSs), 63 structural tRNAs, and six rRNAs were predicted. Among all CDSs, 2,354 were assigned to known protein functions, while the remaining 526 were identified as hypothetical proteins. Additionally, there are 334 RAST subsystems represented in the genome, which represent only 41% of the assigned sequences.

Genomic analysis using BAGEL3 (6), RAST (3), and BLAST demonstrated the presence of putative bacteriocin class II clusters in contig_146 (accession no. LUHN01000033.1) and in contig_176 (accession no. LUHN01000051.1). The contiguous 13,678-bp sequence of contig_146 revealed four operon-like structures (i.e., *plnABCD, plnEFI*, *plnJKLR* and *plnMNOP*) related to the production of the class IIb bacteriocins plantaricin E/F and J/K, as described for both *L. plantarum* C11 (7) and the reference genome *L. plantarum* WSCF1. On the other hand, *plnMNOP* and *plnGHSTUV* were detected, and *plnGH* is thought to encode subunits of an ABC transporter that secretes and processes the bacteriocin precursors. The functions of PlnO, PlnN, PlnR, and PlnSTUV are not known. In *L. plantarum* MPL16, the *plnGHSTUV* operon is located in contig_176, and BLAST analysis revealed the absence of the *plnS* gene.

The draft genome sequence of *L. plantarum* MPL16 will be useful for further studies of specific genetic features of this strain and for its biotechnological application in the development of novel functional feeds using wakame. In addition, genome information will be of value for understanding the mechanisms of the strain’s immunobiotic properties in the porcine host.

### Accession number(s).

This whole-genome shotgun project has been deposited at DDBJ/EMBL/GenBank under the accession number LUHN00000000. The version described in this paper is the first version, LUHN00000000.1.
